# Supplementary material for: Content-rich biological network constructed by mining PubMed abstracts
Source: BMC Bioinformatics. 2004 Oct 8;5:147. doi: 10.1186/1471-2105-5-147 (PMC528731; doi:10.1186/1471-2105-5-147)
Supplement: Additional File 2 — The original results of the above study (non-essential files are deleted to keep the file size under the limit set by BMC bioinformatics). [file 1471-2105-5-147-S2.bz2 › chilibotAdditionalFile2/dip05/165ID8599116E572/html/AKAP5_PRKCA.html]

 


 **AKAP5** and **PRKCA** 
  
Found 15 abstracts in PubMed, retrieved 05.  
 

 What does Google say? 
 PDF only 
| .edu only 

---

- J Pharmacol Exp Ther, 2002   **Calcium and protein kinase C PKC related kinase mediate alpha 1A adrenergic receptor stimulated activation of phospholipase D in rat 1 cells, independent of PKC.** .
  A previous study conducted in rat 1 cells expressing alpha 1A adrenergic receptors showed that phenylephrine PHE stimulates phospholipase D PLD activity.
  This study was conducted to determine the contribution of protein kinase C PKC to PHE induced PLD activation in these cells.
  PKC inhibitors bisindolylmaleimide BIM I and Ro 31 8220, but not Gö 6976 or a pseudosubstrate peptide inhibitor of PKCalpha, decreased PLD activity and arachidonic acid release elicited by PHE.
  However, antisense oligonucleotides directed against PKC alpha **[PRKCA]**, delta, epsilon, and eta reduced PKC isoform levels by about 80% but failed to alter PHE induced PLD activation, indicating that these PKC isoforms are not involved in PLD activation elicited by alpha1A adrenergic receptor stimulation.
  Ectopic expression of a kinase **[AKAP5]** deficient mutant of the PKC related kinase PKN significantly attenuated PHE induced PLD activation.
  On the other hand, BIM I and Ro 31 8220 blocked PHE mediated increase in intracellular Ca2 but Gö 6976 and the peptide inhibitor did not.
  In the absence of extracellular Ca2 , PHE failed to increase PLD activity.
  These results indicate that alpha1A adrenergic receptor stimulated PLD activation is mediated by a mechanism independent of PKCalpha, delta, epsilon, and eta, but dependent on a PKC related kinase, PKN.
  Moreover, PKC inhibitors BIM I and Ro 31 8220 block PHE induced PLD activity by inhibiting calcium signal.
  Caution should be used in interpreting the data obtained with PKC inhibitors in vivo.

  - EMBO J, 2001   **Ezrin is a downstream effector of trafficking PKC integrin complexes involved in the control of cell motility.** .
    Protein kinase C PKC alpha **[PRKCA]** has been implicated in beta1 integrin mediated cell migration.
    Stable expression of PKCalpha is shown here to enhance wound closure.
    This PKC driven migratory response directly correlates with increased C terminal threonine phosphorylation of ezrin moesin radixin ERM at the wound edge.
    Both the wound migratory response and ERM phosphorylation are dependent upon the catalytic function of PKC and are susceptible to inhibition by phosphatidylinositol 3 kinase blockade.
    Upon phorbol 1 3 dibutyrate stimulation, green fluorescent protein PKCalpha and beta1 integrins co sediment with ERM proteins in low density sucrose gradient fractions that are enriched in transferrin receptors.
    Using fluorescence lifetime imaging microscopy, PKCalpha is shown to form a molecular complex with ezrin, and the PKC co precipitated endogenous ERM is hyperphosphorylated at the C terminal threonine residue,i.e. activated.
    Electron microscopy showed an enrichment of both proteins in plasma membrane protrusions.
    Finally, overexpression of the C terminal threonine phosphorylation site mutant of ezrin has a dominant inhibitory effect on PKCalpha induced cell migration.
    We provide the first evidence that PKCalpha or a PKCalpha associated serine threonine kinase can phosphorylate the ERM C terminal threonine residue within a kinase **[AKAP5]** ezrin molecular complex in vivo.

    - Traffic, 2000   **Rab2 requires PKC iota lambda to recruit beta COP for vesicle formation.** .
      The small GTPase Rab2 initiates the recruitment of soluble components necessary for protein sorting and recycling from pre Golgi intermediates.
      Our previous studies showed that Rab2 required protein kinase C PKC or a PKC like protein to recruit beta COP to membrane Tisdale EJ, Jackson M.
      Rab2 protein enhances coatomer recruitment to pre Golgi intermediates.
      J Biol Chem 1998;273 17269 17277 .
      We investigated the role of PKC in Rab2 function by first determining the active isoform that associates with membranes used in our assay.
      Western blot analysis detected three isoforms PKC alpha **[PRKCA]**, gamma and iota lambda.
      A quantitative binding assay was used to measure recruitment of these kinases when incubated with Rab2.
      Only PKC iota lambda translocated to membrane in a dose dependent manner.
      Microsomes treated with anti PKC iota lambda lost the ability to bind beta COP, suggesting that Rab2 requires PKC iota lambda for beta COP recruitment.
      The recruitment of beta COP to membranes is not regulated by PKC iota lambda kinase activity.
      However, PKC iota lambda activity was necessary for Rab2 mediated vesicle budding.
      We found that the addition of either a kinase **[AKAP5]** deficient PKC iota lambda mutant or atypical PKC pseudosubstrate peptide to the binding assay drastically reduced vesicle formation.
      These data suggest that Rab2 causes translocation of PKC iota lambda to vesicular tubular clusters VTCs , which promotes the recruitment of COPI to generate retrograde transport vesicles.

      - J Biol Chem, 2001   **Phosphorylation of tau is regulated by PKN.** .
        For the phosphorylation state of microtubule associated protein, tau plays a pivotal role in regulating microtubule networks in neurons.
        Tau promotes the assembly and stabilization of microtubules.
        The potential for tau to bind to microtubules is down regulated after local phosphorylation.
        When we investigated the effects of PKN activation on tau phosphorylation, we found that PKN triggers disruption of the microtubule array both in vitro and in vivo and predominantly phosphorylates tau in microtubule binding domains MBDs .
        PKN has a catalytic domain highly homologous to protein kinase C PKC , a kinase **[AKAP5]** that phosphorylates Ser 313 = Ser 324, the number used in this study in MBDs.
        Thus, we identified the phosphorylation sites of PKN and PKC subtypes PKC alpha **[PRKCA]**, betaI, betaII, gamma, delta, epsilon, zeta, and lambda in MBDs.
        PKN phosphorylates Ser 258, Ser 320, and Ser 352, although all PKC subtypes phosphorylate Ser 258, Ser 293, Ser 324, and Ser 352.
        There is a PKN specific phosphorylation site, Ser 320, in MBDs.
        HIA3, a novel phosphorylation dependent antibody recognizing phosphorylated tau at Ser 320, showed immunoreactivity in Chinese hamster ovary cells expressing tau and the active form of PKN, but not in Chinese hamster ovary cells expressing tau and the inactive form of PKN.
        The immunoreactivity for phosphorylated tau at Ser 320 increased in the presence of a phosphatase inhibitor, FK506 treatment, which means that calcineurin protein phosphatase 2B may be involved in dephosphorylating tau at Ser 320 site.
        We also noted that PKN reduces the phosphorylation recognized by the phosphorylation dependent antibodies AT8, AT180, and AT270 in vivo.
        Thus PKN serves as a regulator of microtubules by specific phosphorylation of tau, which leads to disruption of tubulin assembly.

        - Endocrinology, 1998   **Modulation of estrogen receptor levels in mouse uterus by protein kinase C isoenzymes.** .
          We have recently shown that protein kinase C PKC modifies estrogen receptor ER binding and modulates the responsiveness to estrogens in a clonal osteoblast like cell line stably transfected with the ER.
          The purpose of the present study was to determine whether the interaction observed between the ER and PKC signaling in these cells occurs in additional estrogen target organs, such as the uterus.
          When uteri were incubated for 2 h with increasing concentrations of a kinase **[AKAP5]** inhibitor H7 , ER binding was enhanced in a dose dependent manner.
          Stimulation of PKC with phorbol ester reduced PKC activity levels, but increased ER binding.
          Interestingly, the changes in binding appeared to be due primarily to alterations in cytosolic ER levels, as binding in the nuclear fraction was minimally enhanced.
          When levels of ER messenger RNA were evaluated by Northern blot analysis, no differences were observed among the H7 or 12 O tetradecanoylphorbol 13 acetate TPA treated and untreated groups.
          Western blot analysis demonstrated that levels of ER cytosolic protein in the H7 , TPA , and staurosporine treated groups were increased relative to those in the untreated controls.
          When uteri were incubated with diethylstilbestrol in the presence of either H7 or TPA, no change in cytosolic ER levels was found, suggesting that only unoccupied ERs are responsive to modulation by PKC.
          Western blotting of the various PKC isoforms indicated that although PKC alpha **[PRKCA]**, beta1, betaII, delta, and zeta are expressed in the uterus, only PKC alpha **[PRKCA]** and beta1 are translocated from the soluble to the particulate fraction and then degraded after phorbol ester stimulation.
          Hence, one or both of these latter PKC isoforms may regulate cytosolic ER levels.
          Collectively, these data indicate that PKC may play an important role in the modulation of uterine ER levels.
          PKC may exert its effect on the ER at some posttranscriptional or posttranslational step.
          Finally, our results show that an ER PKC interaction occurs in a whole organ such as the uterus.
          this interaction may be important in the regulation of the ER activity in a variety of estrogen responsive tissues.
